# Giant Mesenteric Cyst: Diagnostic Challenges and Surgical Management in a Clinical Case

**DOI:** 10.7759/cureus.88010

**Published:** 2025-07-15

**Authors:** Nora Lis Flores Olmos, Gerardo Evaristo Mendez, Daniel Eduardo Luna López, Roberto Armando Gutiérrez Ceballos, Iván Botello Ramírez

**Affiliations:** 1 Department of General Surgery, Dr. Valentín Gómez Farías Regional Hospital, Institute for Social Security and Services for State Workers, Zapopan, MEX; 2 Department of General Surgery, Hospital General De Occidente, Zapopan, MEX

**Keywords:** abdominal mass, abdominal pain, exploratory laparotomy, mesenteric cyst, surgical resection

## Abstract

Mesenteric cysts are rare intra-abdominal cystic lesions of uncertain etiology, often diagnosed incidentally due to their variable and nonspecific clinical presentation. We present the case of a 36-year-old male patient with chronic abdominal pain and distension, in whom computed tomography (CT) revealed a giant mesenteric cyst. Complete surgical resection was performed via laparotomy, and histopathological examination confirmed its benign nature. The patient had a favorable postoperative course without complications. Diagnosis of these lesions requires a high index of suspicion and imaging support, while complete excision remains the treatment of choice to prevent recurrence. Despite their rarity, mesenteric cysts should be considered in the differential diagnosis of abdominal masses, with timely multidisciplinary management essential to optimize clinical outcomes.

## Introduction

The term mesenteric cyst refers to a heterogeneous group of cystic lesions of various origins that arise in the retroperitoneum or abdomen. The first known description was made by the Italian anatomist Benevieni in 1507 during an autopsy of an eight-year-old child. Surgical excision of such a lesion was first performed by Tillaux in 1880 [1].

Mesenteric cysts are rare, benign intra-abdominal tumors that can occur at any age, with a documented female-to-male ratio of 2:1 and an estimated incidence of one in 100000 adults and one in 20000 children [2]. Although they are generally benign, they carry a reported malignant transformation risk of approximately 3% [3].

Their etiology remains uncertain. The most widely accepted hypothesis suggests that they result from benign ectopic lymphatic proliferation within the mesentery, not connected to the remainder of the lymphatic system [4].

These lesions often present with variable and nonspecific symptoms, and up to 40% of cases are discovered incidentally during physical examinations or imaging studies performed for unrelated reasons [3]. Many patients are completely asymptomatic, especially those with small cysts. When symptoms are present, they are typically nonspecific gastrointestinal complaints, such as abdominal pain, nausea, vomiting, constipation, or diarrhea [5].

The clinical presentation depends largely on cyst size and the patient’s age. In children, mesenteric cysts may mimic appendicitis, whereas adults are more likely to be asymptomatic. A palpable abdominal mass is identified in up to 61% of cases [6]. Mesenteric cysts can occur anywhere along the mesentery, from the duodenum to the rectum, and may extend from the mesenteric root to the retroperitoneum. More than half are located in the small bowel mesentery, most frequently in the ileum [4,7].

## Case presentation

A 36-year-old male patient without chronic degenerative diseases or previous surgeries presented approximately six months prior to hospitalization with chronic constipation, abdominal distension, and intermittent generalized abdominal pain. On the day of admission, symptoms worsened, with increased abdominal pain predominantly in the hypogastrium, radiating to the lumbar region.

**Figure 1:**
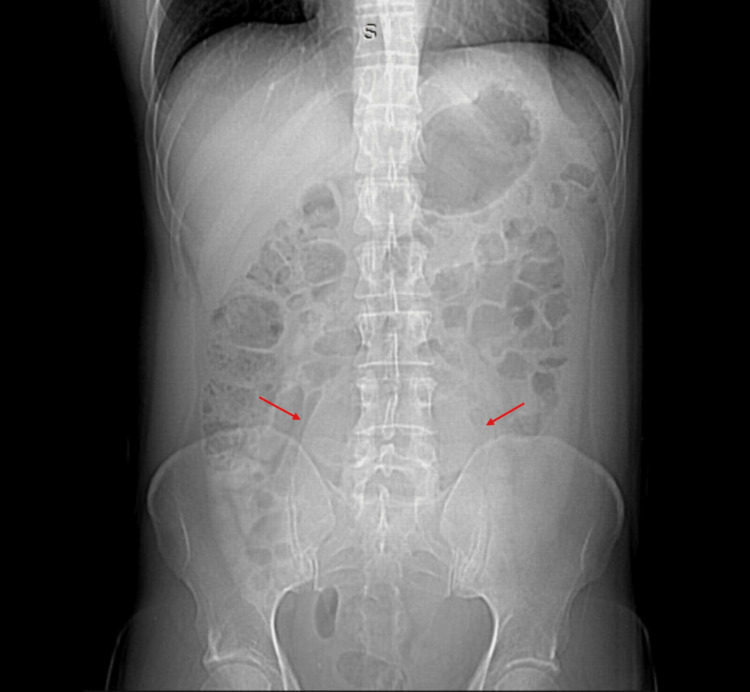
Plain abdominal radiograph showing a pelvic space-occupying lesion displacing the intestinal loops

**Figure 2:**
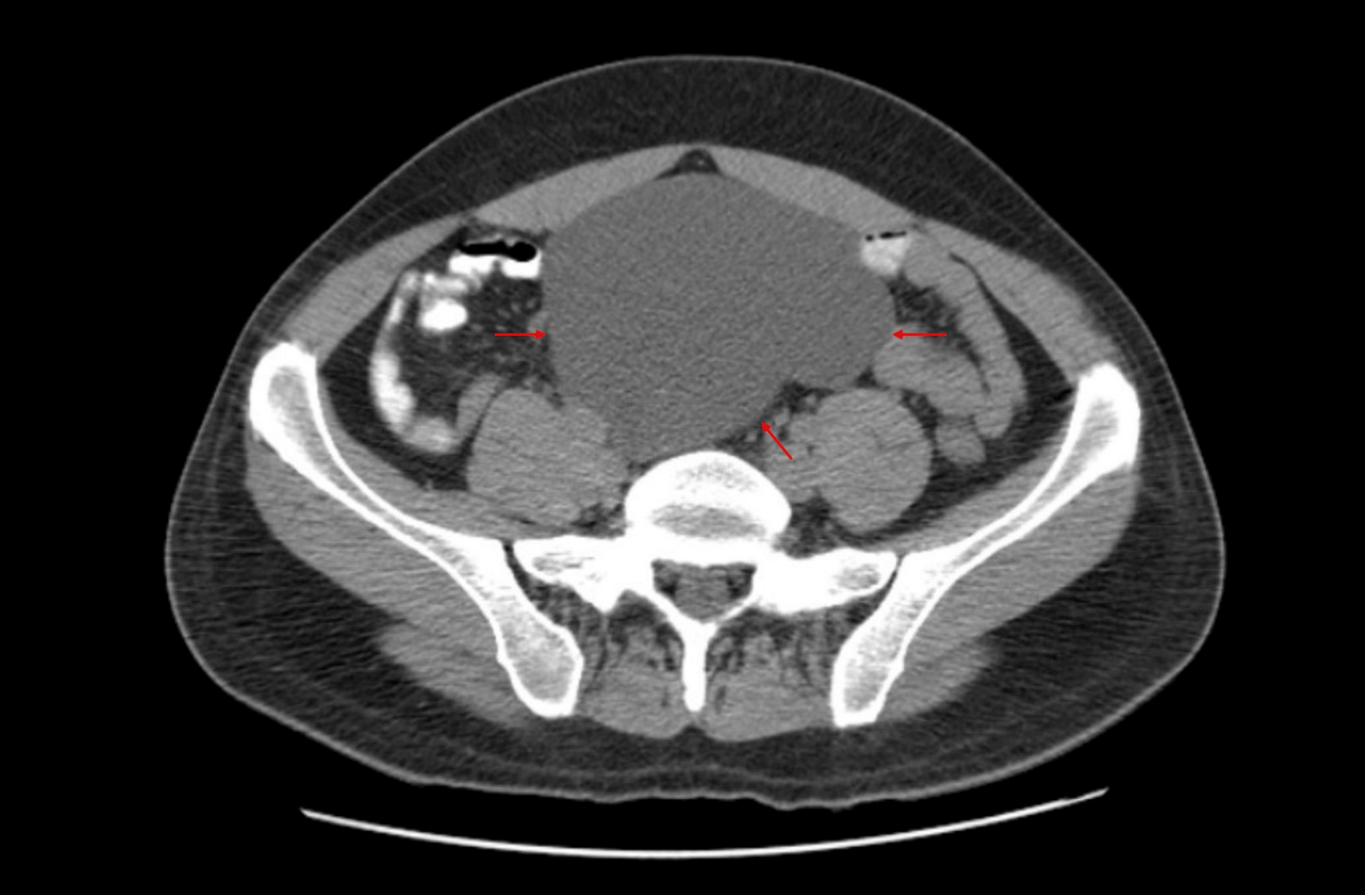
Contrast-enhanced CT scan, axial view, showing a mesenteric cystic mass with homogeneous liquid isodensity and wall enhancement after contrast administration; measures 11.9 cm × 9 cm × 20 cm CT: computed tomography

Upon admission to the emergency department, there were no signs of an acute abdomen or systemic inflammatory response. A plain abdominal X-ray showed an occupying lesion in the pelvic cavity displacing intestinal loops (Figure 1), leading to the request for contrast-enhanced computed tomography (CT). Imaging revealed a mesenteric cystic mass in the mesogastrium and hypogastrium with homogeneous liquid isodensity and contrast-enhanced wall, measuring 11.9 cm x 9 cm x 20 cm (Figure 2). The patient was evaluated by our service and scheduled for surgical intervention

**Figure 3:**
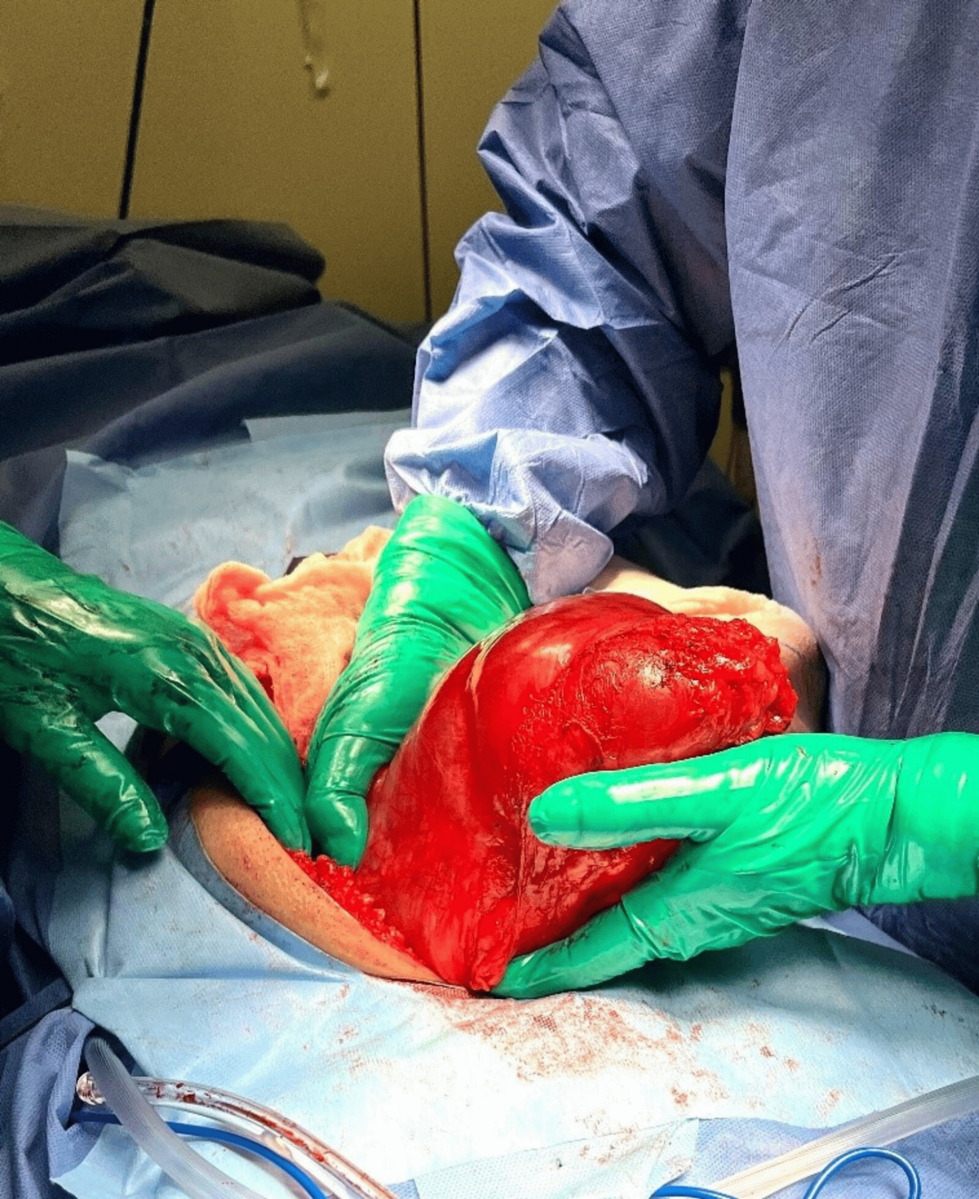
A giant mesenteric cyst is observed

A diagnostic laparotomy revealed a large cystic peritoneal mass firmly adherent to the posterior abdominal wall, extending from the umbilical region to the rectum, measuring approximately 15 cm × 20 cm. Both the small and large intestines were free of adhesions, and there was no evidence of bladder infiltration (Figure 3).

**Figure 4:**
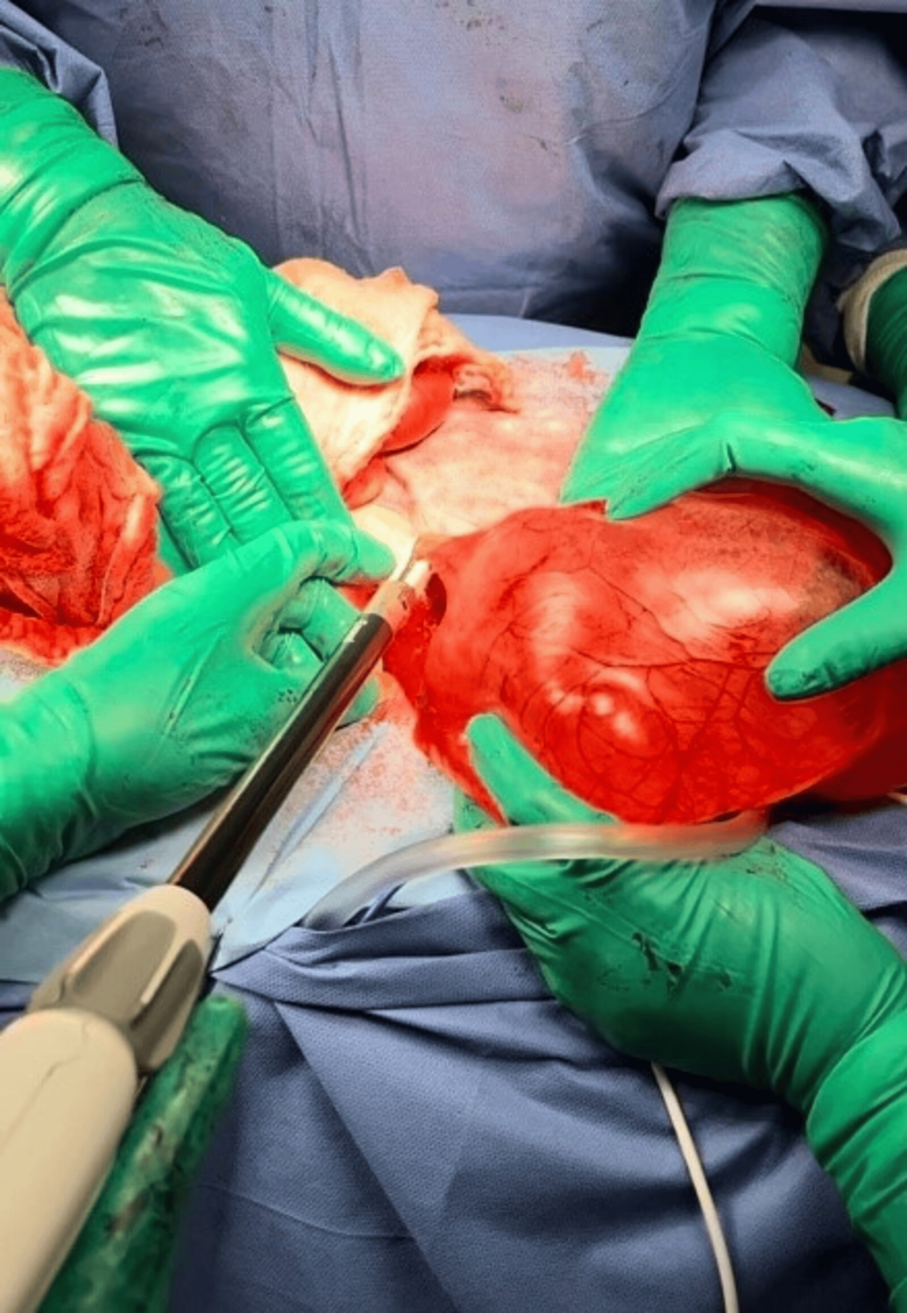
Bipolar energy is used for effective vessel sealing

**Figure 5:**
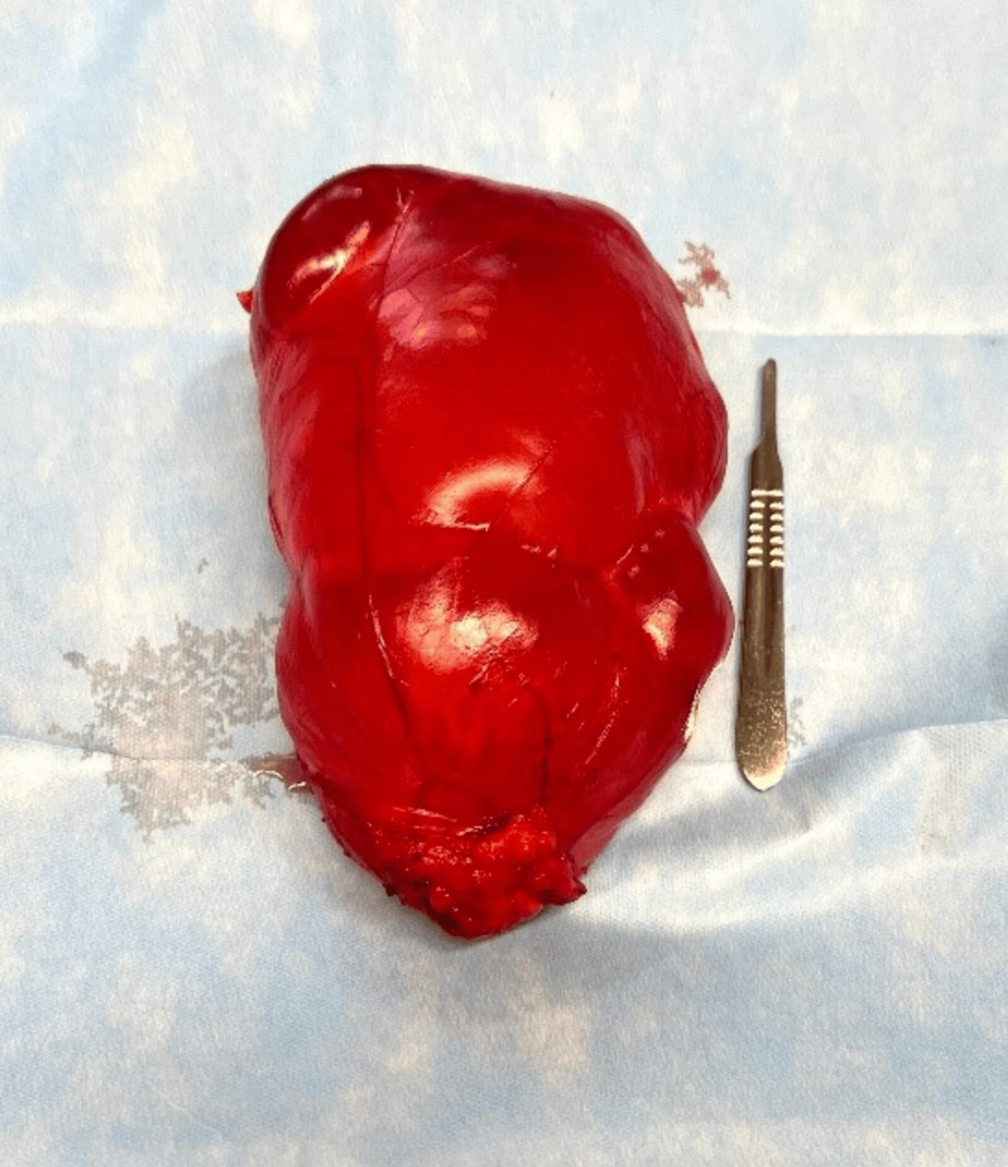
Pathological specimen

Dissection and mobilization were initiated, including a segment of the posterior abdominal wall within the specimen to ensure complete excision of the cystic peritoneal mass. Electrocautery and a bipolar energy device (Enseal) were used for hemostasis and vessel sealing (Figure 4). The entire specimen was excised en bloc and submitted for histopathological analysis (Figure 5).

The patient was admitted to the post-anesthesia care unit and initiated on a liquid diet, which was well tolerated and advanced the following day. The postoperative course was uneventful, and the patient was discharged on postoperative day four with scheduled follow-up in the general surgery outpatient clinic. Histopathological analysis subsequently confirmed a multiloculated mesenteric cyst with benign features.

## Discussion

Mesenteric cysts are uncommon lesions that often pose a diagnostic challenge due to their frequently asymptomatic nature and wide variability in clinical presentation. Although they have been described since the 16^th^ century, their pathogenesis remains controversial [2]. These cysts are believed to originate from congenital anomalies of lymphatic tissue, trauma, or infectious etiologies. Malignant transformation is rare, accounting for less than 3% of reported cases, and tends to occur more often in adults, frequently in association with sarcomatous changes [4,7].

These cysts may arise anywhere along the mesentery but are predominantly found in the small bowel mesentery, especially the ileum [2,4]. This localization is clinically significant, as compression of adjacent structures can produce nonspecific abdominal symptoms that range from chronic discomfort to acute abdomen, thereby complicating timely diagnosis [8]. In the present case, the patient exhibited abdominal pain and nausea, symptoms consistent with reports indicating that up to 10% of patients with mesenteric cysts may present with acute abdomen [7,8].

Imaging studies, primarily ultrasound and CT, constitute the cornerstone for the diagnosis of mesenteric cysts [2,4]. Nonetheless, definitive diagnosis is occasionally only established intraoperatively due to the complex anatomy and variable presentation of these masses [4,7]. 

Complete surgical excision remains the treatment of choice to prevent recurrence and potential complications [4,7]. While laparoscopic surgery is preferred given its association with reduced morbidity, large cysts or those with adherence to neighboring structures often require open surgical approaches [2]. Partial excision or simple drainage is discouraged due to the high rates of recurrence associated with these techniques [2,8].

Despite their rarity, prompt recognition and management of mesenteric cysts are essential to avoid serious adverse outcomes. The broad clinical spectrum and diagnostic challenges underscore the importance of comprehensive clinical assessment and a multidisciplinary approach to treatment.

## Conclusions

Mesenteric cysts, although rare, pose a considerable diagnostic challenge due to their variable and frequently nonspecific clinical manifestations. Their predominant localization within the small bowel mesentery, coupled with the potential to induce acute abdominal symptoms, necessitates their inclusion in the differential diagnosis of atypical abdominal presentations. Timely diagnosis, supported by imaging modalities such as CT, is essential for appropriate surgical planning, with definitive confirmation often achieved during the operative procedure.

Complete surgical excision remains the gold standard treatment. A laparoscopic approach is preferred when anatomical conditions are favorable, as it offers reduced morbidity and recurrence rates; however, open surgery continues to be a safe and effective alternative when laparoscopy is not feasible. Ultimately, a multidisciplinary approach is key to optimizing patient outcomes and highlights the importance of maintaining a high index of suspicion when faced with incidental findings or nonspecific abdominal complaints.
